# Irriman Platform: Enhancing Farming Sustainability through Cloud Computing Techniques for Irrigation Management

**DOI:** 10.3390/s22010228

**Published:** 2021-12-29

**Authors:** Manuel Forcén-Muñoz, Nieves Pavón-Pulido, Juan Antonio López-Riquelme, Abdelmalek Temnani-Rajjaf, Pablo Berríos, Raul Morais, Alejandro Pérez-Pastor

**Affiliations:** 1 Agronomic Engineering Department, Agronomic Engineering Technical School, Alfonso XIII Campus, Technical University of Cartagena, 30203 Cartagena, Spain; manuel.forcen@edu.upct.es (M.F.-M.); abdelmalek.temnani@edu.upct.es (A.T.-R.); pablo.berrios@edu.upct.es (P.B.); alex.perez-pastor@upct.es (A.P.-P.); 2 Automation, Electrical Engineering and Electronic Technology Department, Industrial Engineering Technical School, Muralla del Mar Campus, Technical University of Cartagena, 30202 Cartagena, Spain; jantonio.lopez@upct.es; 3 INESC TEC—Institute for Systems and Computer Engineering, Technology and Science, Pólo da UTAD, University of Trás-os-Montes e Alto Douro, 5000-801 Vila Real, Portugal; rmorais@utad.pt

**Keywords:** intelligent irrigation, GIS, cloud systems, precision agriculture, crop sustainability

## Abstract

Crop sustainability is essential for balancing economic development and environmental care, mainly in strong and very competitive regions in the agri-food sector, such as the Region of Murcia in Spain, considered to be the orchard of Europe, despite being a semi-arid area with an important scarcity of fresh water. In this region, farmers apply efficient techniques to minimize supplies and maximize quality and productivity; however, the effects of climate change and the degradation of significant natural environments, such as, the “Mar Menor”, the most extent saltwater lagoon of Europe, threatened by resources overexploitation, lead to the search of even better irrigation management techniques to avoid certain effects which could damage the quaternary aquifer connected to such lagoon. This paper describes the Irriman Platform, a system based on Cloud Computing techniques, which includes low-cost wireless data loggers, capable of acquiring data from a wide range of agronomic sensors, and a novel software architecture for safely storing and processing such information, making crop monitoring and irrigation management easier. The proposed platform helps agronomists to optimize irrigation procedures through a usable web-based tool which allows them to elaborate irrigation plans and to evaluate their effectiveness over crops. The system has been deployed in a large number of representative crops, located along near 50,000 ha of the surface, during several phenological cycles. Results demonstrate that the system enables crop monitoring and irrigation optimization, and makes interaction between farmers and agronomists easier.

## 1. Introduction

Multiple worrying events related to climate change, which already have a great influence in most ecosystems around the world, are currently happening [1]. Although geological records demonstrate that climate variations have occurred in the past without human intervention, caused by many natural factors, nowadays, most experts agree that such current events are partly a consequence of human activity. On the other hand, population growth [2] is a fact; human beings have basic needs to be covered, mainly related to food production and consumption, and finding the balance between food production and environmental sustainability is often hard, since many social and economic aspects should be considered [3].

The “Mar Menor”, considered to be the largest saltwater lagoon in Europe [4], located at the southeast part of the Iberian Peninsula, on the Mediterranean coast, is an example of degradation of a valuable ecosystem due to multiple causes directly related to extreme climate events and human activity. Experts warn that poor management of the land surrounding the lagoon is a major problem which should be addressed [5,6]. Enhancing urban planning and sanitation networks, and avoiding pollution generated by intensive farming are required actions to carry out.

Agribusiness in the Region of Murcia (Spain), where the mentioned lagoon is located, is particularly relevant from a social and economic point of view, becoming considered as the orchard of Europe, despite being a semi-arid area with an important scarcity of fresh water. According to the latest figures published by the regional government, farmland occupies a large area around 600,000 ha, where 68% is dedicated to dry land crops and the rest to irrigation. In particular, “Campo de Cartagena” is a specific area of around 50,000 ha, with important irrigation crops surrounding the lagoon.

Water scarcity and environmental challenge of protecting such a valuable ecosystem have led farmers to a complex scenario, where it is necessary to apply efficient techniques to minimize supplies, maximize crop quality and productivity and avoid any kind of pollution which could damage the lagoon and its surrounding area.

Specifically, the state of the “Campo de Cartagena” quaternary aquifer has been studied by experts (at the request of the Spanish Ecological Transition and Demographic Challenge Ministry), concluding that a large number of nitrates is present in its waters [5]. This situation involves the execution of more appropriate agricultural practices, from an environmental perspective, aimed to reduce the dosage of nitrates. Such practices need to be properly monitored and controlled and, in this context, the use of techniques based on Information and Communication Technologies (ICTs) is crucial.

Precision Agriculture (PA) strategies have demonstrated that it is possible to acquire, process and analyze temporal, spatial an individual data, combining them with other information to support management decisions aimed to improve resource use efficiency, productivity, quality, profitability and sustainability of agricultural production [7]. ICTs make field level crop management more operational and easier [8], since data gathering is often carried out by using specific electronic instrumentation deployed in crops. Such devices allow ambient, plant and soil information to be sensed and properly sent to processing elements, capable of generating knowledge useful for Decision Support Systems (DSSs), in the context of crop management [9].

A typical PA approach could include drones and satellite imagery for determining variable-rate seeding, creating yield maps of areas according to their productivity or calculating NDVIs (Normalized Difference Vegetation Indexes), at farm level [10]. The Internet of Things (IoT), understood as a network of physical electronic devices for data collection and, eventually, aggregation, is often used in the context of PA. In particular, specific agronomic sensors can be deployed and connected to electronic devices, capable of reading and adapting the resulting signal to a digital format. Such information is then sent over a local or global communication network and remotely stored in a server for undertaking posterior analytics. Gathered data and their analysis [11] can be presented to users through applications running in personal computers (PCs) or smartphones [12]. Thus, farmers and other stakeholders could easily control crop productivity, avoid or minimize pest effects or ensure that certain environmental conditions are within established limits. Furthermore, Artificial Intelligence techniques could also enable the installation of nearly autonomous fertigation systems, where actions are carried out by automatized actuators, which execute actions from the outputs generated by the mentioned DSSs.

A significant number of research works related to PA are found in the state of the art. Specifically, the systems that use Wireless Sensor Networks (WSNs) [8], have been widely tested in diverse crops, under different conditions [13,14,15,16]. Some of the works are focused on evaluating the network structure in terms of coverage range, throughput, latency or consumption, among other common parameters related to electronic design and communication techniques. Others study the benefits of accurately monitoring crops at different levels (soil, plant and ambient), from a strictly agronomic point of view [17]. In these cases, productivity is often considered one of the main parameters to evaluate.

Most researchers present their results after evaluating the deployed system in a specific controlled crop. In general, the outline of such works includes the WSN design (often a heterogeneous one), with several end devices and one or several local electronic devices acting as sinks, which send collected data to a remote server via Internet. There, the information is processed and presented to end users [18,19,20].

Wired solutions, such as commercial data loggers (for example, Campbell Scientific-based systems), are still used in large agricultural exploitations for connecting typical manufactured agronomic sensors, but these solutions are often expensive, data are locally stored, specialized staff is needed for data acquisition and interpretation and this kind of system is not low powered.

On the other hand, Cloud-based farm management systems have emerged as a solution for farmers, with the purpose of providing them frameworks that allow the interconnection among services developed by different service providers and tailored to their needs [21].

However, neither in the literature nor the market, it is easy to find crop monitoring systems that collect and combine information from multiple sources (drones, remote sensing, soil, ambient and plant sensors and meteorological information), store data by using Cloud and Edge Computing-based techniques and provide usable tools for information interpretation, ready to be used from a PC or smartphone, both by farmers and Agronomic Engineering experts.

This paper describes the Irriman Platform, a novel crop monitoring system, including a GIS (Geographical Information System) tool, which uses Cloud Computing techniques, for providing all the necessary features that allow farmers and experts to apply and evaluate different irrigation and fertigation strategies [22] in any kind of crop, with soil and plant sensors installed. The system has already been deployed in a wide range of crops in agricultural exploitations located at the “Campo de Cartagena” area. The DSS included in the platform is aimed to optimize irrigation, with the purpose of saving fresh water, maintaining or even increasing productivity and mainly protecting the quaternary aquifer connected to the “Mar Menor”, avoiding the process of leaching, as much as possible. Leaching control is essential because the number of nitrates that leach from a soil depends on the amount of water that goes through the soil, together with the number of nitrates in the soil when water drains through and out of the soil profile [23]. The pollution of the lagoon with fertilizer nitrates from agricultural techniques, through the connection with the aquifer, happens because phytoplankton is fertilized and, when its reproduction is indiscriminate, it hides sun light, and the seagrass beds die, unable to carry out photosynthesis. Therefore, if farmers are capable of accessing information about how the soil dynamics works through the Irriman Platform, it is possible to adapt the irrigation procedure for avoiding leaching and, consequently, the aquifer is protected from this kind of pollution, maintaining farmers’ lifestyles and contributing to economic and social development in a sustainable manner.

The outline of the paper is as follows. Section 2 describes the material and methods used and designed for the Irriman Platform development. Section 3 details how users could access the Irriman Platform through a WEB application. Moreover, the performance of the platform is tested in depth, demonstrating that it is a useful and usable tool for helping farmers to make their irrigation methods environmentally compatible. Cloud Computing techniques are specifically evaluated, in terms of latency, reliability and economic cost, among others. A discussion about the proposed system is presented in Section 4. Finally, conclusions are addressed in Section 5.

## 2. Materials and Methods

In this section, the architecture of the proposed system is detailed. Hardware and software components are described, including the devices deployed at the lowest level in crops, together with the firmware designed for data acquisition, the communication protocol for sharing such data, and the selected Cloud Computing techniques which enable information storage and processing and knowledge extraction and presentation. The software architecture follows the known client–server model, but the backend developed in the “server side” is deployed as a set of Cloud components running in the Cloud infrastructure provided by Google.

### 2.1. The Irriman Platform Architecture

**Figure 1:**
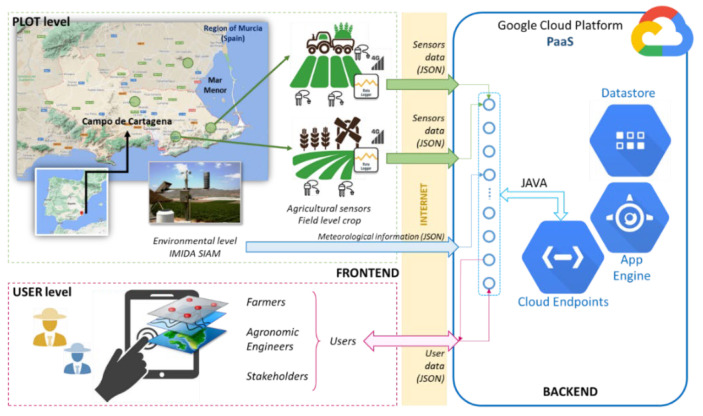
Global architecture of the Irriman Platform. The frontend consists of two levels: PLOT and USER. The backend is implemented as a set of modules running on the Google Cloud Platform. Data loggers acquire information from agricultural sensors and send them to the backend by using the HTTPS protocol and JSON messages as payload. Users could interact with the backend through a WEB application that sends and receives JSON messages by also using the HTTPS protocol.

Figure 1 shows the full architecture of the Irriman Platform. As with any system based on the Client/Server model, there are software components acting in the backend, running, in this case, as a part of the Google Cloud Platform. Such components have been developed using tools delivered by the Platform as a Service (PaaS) cloud layer that Google provides. The frontend consists of software modules that interact with the backend through an API (Application Programming Interface) REST (Representational State Transfer), by using the HTTPS (HyperText Transfer Protocol Secure), communication protocol for sharing information coded as JSON (JavaScript Object Notation), messages. Data loggers connect all the agricultural sensors that gather data at the crop level. The program running in the data logger acts as a client. It processes the acquired information, codes it as JSON messages, and sends it to the Cloud through the mobile network, by using the same API REST as the WEB application that allows users to remotely interact with the platform. This WEB application also works as a client; the frontend includes software modules in both the PLOT level and USER level. The whole system could be considered as a distributed application, where different software modules and interfaces request services to the backend and provide suitable responses to different clients. Thus, users use services related to agronomic data visualization, together with aggregated information useful to know the behaviors of soil; and plant and data loggers installed at crop level use the backend services for uploading data measured by agronomic sensors of different kind. These devices send readings at a specific rate (now, most of the installed ones send data every 10 min, which could be considered as an appropriate rate to obtain time series that reflect the behavior of the soil, according to the water content received by the irrigation system installed in each crop or because of rainfall).

Environmental information is obtained from the IMIDA (Instituto Murciano de Investigación y Desarrollo Agrario y Medioambiental), SIAM (Sistema de Información Agrícola de Murcia). SIAM is a net of 47 automatic stations (32 from IMIDA and 15 from other Spanish official institutions), mostly installed in private agricultural exploitations, whose geographical location accomplish a specific set of requirements. A wide set of variables are measured by these stations. The Irriman Platform downloads all the information and it currently uses ET0, average daily temperature and rainfall as essential parameters to be combined with the rest of the data measured by sensors installed at crop level. All the information is properly processed and aggregated, if needed, and displayed by the WEB application designed as one of the mentioned frontends. The other frontend is the low-level software running in each data logger, which simply reads sensors’ measurements and sends the raw data to the Cloud by calling certain backend services.

### 2.2. Hardware and Software Components at the PLOT Level

**Figure 2:**
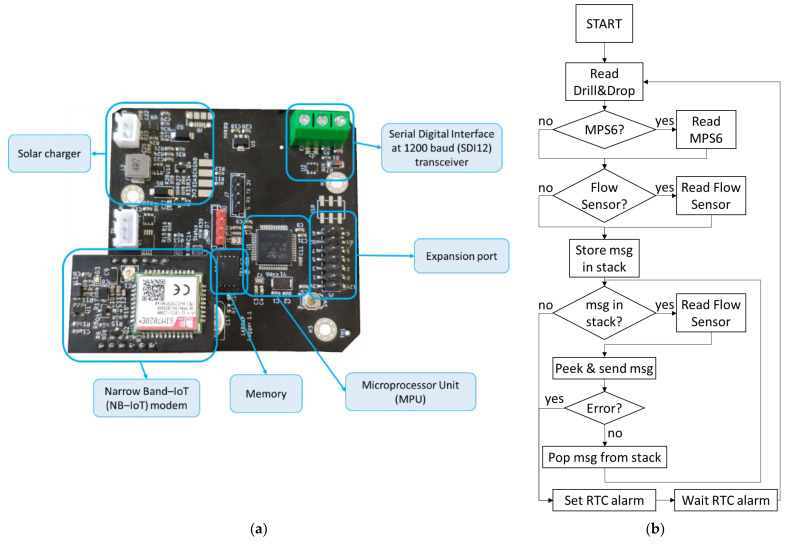
Data logger designed for crop level data acquisition: (**a**) Detail of the electronic design and main hardware components of the data logger. (**b**) Flowchart describing how the firmware runs for gathering, locally storing and sharing read sensed data with the Cloud.

As an essential part of the Irriman Platform, a data logger has been designed for crop level data gathering (see Figure 2). Well-known agronomic sensors could be used for this purpose; consequently, the data logger includes all the necessary electronic modules and interfaces for allowing most types of sensors to be connected.

Figure 2a shows the hardware architecture of the developed device, and Figure 2b details the dataflow that summarizes how the firmware works when the information acquired by each connected sensor is read and subsequently sent through the mobile network, according to the available coverage.

Table 1 shows the description of sensors currently deployed in the plots monitored by using the Irriman Platform. Relevant data are temperature, salinity and volumetric water content found at different depths in the soil. The soil matric potential is also acquired, since it can be considered as a realistic criterion for measuring soil water availability to plants, since this measurement provides information about which water is held by the soil matrix, that is, soil particles and pore space.

In addition, environmental data are also collected, but, in this case, the IMIDA SIAM infrastructure is used by querying a specific external service that provides some important daily parameters, such as, evapotranspiration, also known as ET0, ambient temperature and pluviometry, among others. As mentioned above, the IMIDA SIAM has deployed a wide number of environmental stations throughout the Region of Murcia; it is then possible to obtain the environmental information, linked to a specific crop, by using the data obtained from the closest station to the plot, according to its geolocation, since the Irriman Platform also stores the GPS (Global Positioning System) coordinates for each plot.

As mentioned in Section 2.1, all the data gathered by the sensors and preprocessed by the data logger, and those acquired from other sources (IMIDA SIAM, for example), are directly sent to the Cloud by calling the corresponding designed Endpoint’ service. Such service automatically decodes the received JSON message, it obtains the timestamped information, and it stores it in Google Firestore in Datastore mode.

### 2.3. Data Model and Its Implementation in the Google Datastore

Firestore is the newest version of Datastore provided by Google. It is actually possible to create “Firestore in Datastore mode” database instances, and this is the option selected for storing most data managed by the Irriman Platform, since it offers a NoSQL database built for automatic scaling, high performance and ease of application development with a strongly consistent storage layer and real-time updates.

An equivalence exists between Datastore-based databases and those managed by a RDBMS (Relational Database Management System), because they share common features. The category of object is implemented as a kind in the Datastore, whereas the table is the concept used in a relational database; each kind can have a number of properties corresponding to columns of a single row in a table, and each row is equivalent to an entity, which could be defined as an individual object, considered as the main unit used by Google for quota and cost calculation.

Datastore “is designed to automatically scale to very large data sets allowing applications to maintain high performance as they receive more traffic”; it is very well adapted to store time series (this is important for agriculture applications which should save many timestamped data); queries are actually served by previously built indexes, writes scale by automatically distributing data, if necessary, and reads are also scaled, because the only supported queries are those whose performance scales according to the result set’s size. This means that the performance of a query that returns a number N of entities is the same when searches are carried out over a hundred of over thousands or millions of entities.

**Figure 3:**
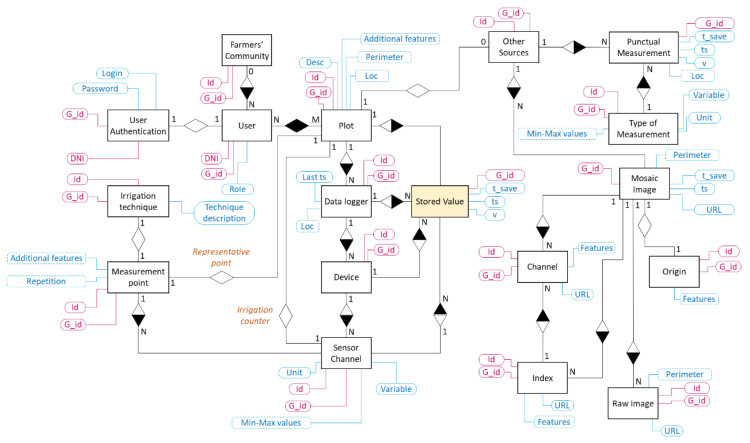
Data model which allows the main entities stored in the Datastore to be defined. Entities are implemented as a Java class whose private attributes are the properties attached to each one. Only the main attributes are represented in this data model. All the entities are created and updated from the WEB application according to the users’ requirements. The Stored Value entities are updated by the data loggers deployed at crop level.

As there are similar characteristics between Datastore and relational databases, it is possible to use well known Entity Relationship Diagrams (ERDs) for database modelling. Figure 3 shows the data model designed for storing the information handled by the Irriman Platform.

The data model has been designed by considering the basic elements which are usually included in ontologies that describe concepts in the PA domain. As the Irriman Platform is mainly aimed to satisfy the needs of many farmers and stakeholders, often belonging to a farmers’ aggrupation, the Farmers’ Community entity is linked to the User entity by a one-to-many relationship. That is, each possible user (farmers, agricultural engineers or technicians and other stakeholders) could belong to zero or an only one specific mentioned aggrupation. Furthermore, each user is univocally identified by his/her DNI (“Documento Nacional del Identidad”, in Spanish). From the DNI, the Google identifier (G_id), is calculated. Such new identifier is used by Google for univocally identifying each Datastore entity (equivalent to a table row in a relational database).

A Plot is the highest-level unit used in the agricultural context. It could be defined as a piece of soil area where one or more crops could be grown. In the designed data model, a specific type of crop is planted in one plot at the same time, although a plot could be reused for new crops in different crop cycles. In this case, when a new crop cycle is started, it is possible to define a new Plot entity to support such new cycle. Thus, it is possible to easily store information from current and past crops grown in the same piece of ground, whose polygon (a set of geolocated points that perimeter the plot), is stored as a list of GPS locations in the Perimeter property. The “Desc” property shows readable information about the plot and the “Additional features” attribute includes some data related to the crop configuration, such as, planting frame dimensions, number of plants per hectare, crop description, variety and pattern and number of irrigation emitters per plant, among others.

Each data logger is installed only in a plot, but one plot could be equipped with several data loggers, consequently, there exists a one-to-many relationship between Plot and Data logger entities. A data logger is located at a specific GPS location, and it should save information about the last timestamp when it sent the last message containing sensed data. This is the reason why the properties “Loc” and “Last ts” are needed.

A data logger allows several devices to be connected by using different interfaces, with the aim of acquiring the information measured by such devices. The firmware, whose flowchart is shown in Figure 2b, collects raw data, performs the necessary calculations for aligning timestamps and values gathered by sensors and, after preprocessing such values, it creates a JSON message ready to be sent to the Cloud by calling the specific Endpoint services through a HTTPs connection. Therefore, there exists a one-to-many relationship between the “Data logger” and “Device” entities, and, also, a one-to-many relationship between the “Device” and “Sensor channel” entities (because each device could be equipped with several sensors measuring the same or different variables).

All these entities, together with their relationships, allow information about deployed devices to be properly stored. Such data uploading is manually carried out from the WEB application when a new plot is included in the system. However, specific timestamped data sent by data loggers should also be saved. For example, probes that collects data about temperature, humidity and salinity usually include different specific sensors located at different depths. A typical probe could measure such values in a range of depths from 10 cm to 90 cm, with sensors each 10 cm. In this case, 9 values of temperature, 9 of humidity and 9 of salinity are acquired. Then, 27 different values should be stored each time for this particular device. The Stored value entity and their relationships with Plot, Data logger, Device and Sensor channel entities enable timestamped data storage. In particular, Stored Value is implemented as a Java class that includes the attributes “ts”, “t_save” and “v” (for saving the timestamp when the value was read, the timestamp when the entity was saved in the Google Cloud and the value itself), and other attributes for storing the references to the mentioned related entities. Thus, it is possible to query time series according to very different filters, for instance, by using timestamp ranges or a specific device or plot, among others.

On the other hand, each Plot is related to the Other Sources entity because it is possible to manually include geolocated punctual measurements, taken by farmers and/or agronomic technicians, or information obtained from images (also geolocated), with a different origin, in particular, those acquired by drones and by hyperspectral imaging satellites. Each mosaic image, which could be the combination of individual raw images, consists of several channels, which are the main data source for indices calculation. As the size of mosaic and raw images, channels and indices exceed the boundaries defined by Google for saving data in the Datastore, such information is stored in an external repository, whose URL is considered as a property in the entities Mosaic Image, Raw Image, Channel and Index.

As the User entity, the rest of entities are univocally referred by a readable identifier, (Id property), which is the base for calculating the G_id. The readable identifier is manually assigned, except for instances of Stored Value. In this case, each readable identifier is calculated by using the “ts” attribute and the reference to the related Sensor channel entity. A similar procedure is applied for instances of Punctual Measurement and Image.

It is possible to query each entity by using this identifier or key, but more complex queries are needed to extract specific information from one or more entities at the same time. Such queries compute its results using one or more indexes, which are updated to reflect the changes made by the application over its entities, “so that the correct results of all queries are available with no further computation needed”. In particular, it is possible to define which properties are indexed in each entity if Java classes and Objectify annotations are used for implementation. In fact, every Java class represents a Datastore type, and every instantiated object can be considered as a Datastore entity. Regarding Datastore indexes, by default a Datastore database automatically predefines an index for each property of each kind, and these indexes are appropriate for simple queries. However, composite indexes are also needed for executing more complex queries, which involve searching and/or ordering by using more than one property. Composite indexes actually index multiple property values per indexed entity, and they should be explicitly defined in an index configuration file, which is deployed with the rest of the application in Google Cloud.

### 2.4. Endpoint Services

The Irriman Platform has been deployed as a GAE (Google App Engine) [24] application that executes services implemented in a Google Endpoint API, remotely used by clients (such as the firmware running in the data loggers, or the WEB application used by users for crop management). With the aim of making the backend development, deployment and supervision easier, Google Cloud Endpoints [25] are used. This framework is defined as a system for managing APIs, enabling developers to “secure, monitor, analyze and set quotas” on each designed API “using the same infrastructure that Google uses for its own APIs”. Moreover, it also provides the tools and libraries needed for generating the REST API used for accessing the GAE application’s services, since Google Endpoint Frameworks handle the low-level communications details of HTTPS requests and responses sent and received. Therefore, when the client sends a specific request to the API, Google Endpoint Frameworks route the request’s URL (Uniform Resource Locator) to the method (written in Java) that will process it. Parameters in the request are passed as JSON structures and they are automatically transformed to Java objects. Information, in the response, is also coded as a JSON message converted from the corresponding Java object.

A wide number of services have been implemented for managing Datastore operations (insertion, deletion and update of entities defined in the Data model described in Section 2.3), and for processing agricultural information with the purpose of obtaining useful knowledge that allows farmers and agronomic engineers and technicians to make decisions aligned with environmental sustainability.

Although some services are only used on rare occasions, for example, creating or updating users, plots or other similar entities, other essential services are very frequently utilised, in particular, one that allows data loggers to send timestamped information and others, such as the WEB application, that both, allow timestamped information in a range to be downloaded, and enable configuration of plots and measurement points to be downloaded and updated.

### 2.5. WEB Application

The Irriman Platform integrates a DSS that uses several kinds of heterogeneous data as an input to a tool that provides some insights how multiple factors are conditioning the soil dynamics. The extracted knowledge helps farmers to optimize irrigation values maintaining productivity and avoiding the leaching process.

Some data are collected by the deployed sensors. Remote sensing, images obtained by drones, meteorological information and punctual measurements, manually taken, are also used as data sources.

**Figure 4:**
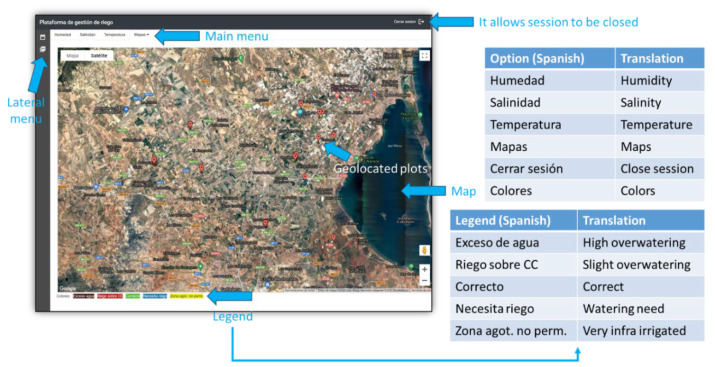
Main screen of the Irriman Platform WEB application. As it is used by farmers and other stakeholders in Spain, text captions are written in Spanish. The translation is shown for the menu options and for the legend that describes how plots are being watered.

Most aggregation and knowledge extraction algorithms are implemented in the client side at the USER level (see Figure 1). This design pattern is aligned with the concept of Edge Computing. Cloud services are used for sending or receiving information suitably packaged as JSON messages, and received data are usually processed by JavaScript methods running in the client machine. Once a collection of data (for example, a time series), is downloaded, it is cached and, consequently, new downloads are not necessary, unless the user explicitly requires it. This kind of interaction between the WEB client and Google Cloud moderates the economic cost, since it helps free quota consumption to be reduced. Furthermore, the performance of the application is better, because graphical representation and aggregation operations are made over the data temporarily stored in the client, without the need for Cloud interaction. Figure 4 shows the main screen of the mentioned WEB application.

### 2.6. GIS WEB Tool

The Irriman Platform also includes a GIS tool, useful for analyzing data obtained from images acquired by using drones and Remote Sensing. It is actually an essential instrument for tracking the soil behavior in both, plots equipped with sensors (as those described in Table 1), and other plots without such equipment.

If sensors are installed, images provide additional data that could be associated with the sensors’ measurements. However, if sensors are missing (due to economic cost or access or coverage problems), images would be the main available data for soil humidity and temperature estimates.

**Figure 5:**
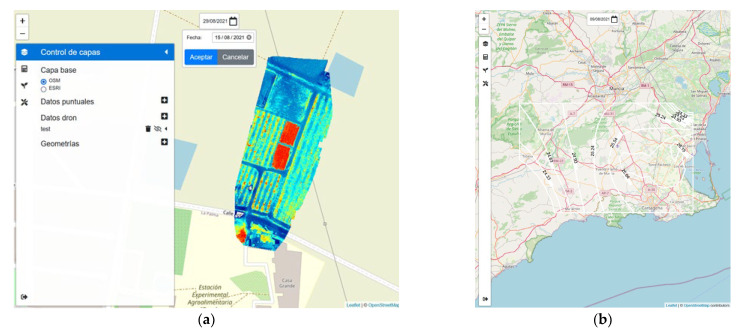
Detail of several functionalities found in the GIS tool. (**a**) Representation of a map with an information layer that contains data calculated from images collected by a drone in a specific interval of time. (**b**) Isoline map that cartographically shows temperature data.

The GIS tool shows additional information acquired by other sources of data, different from sensors connected to the data loggers deployed at soil level. Drones, hyperspectral imaging satellites and devices for taking punctual measurements are included. After collecting all these measurements, they are properly stored in a private repository whose URL is stored in the Datastore. Then, when the GIS tool is selected, all the options for manipulating the images and the measurements, taken by experts, are available through a layered map and a specific contextual menu (see Figure 5). Aggregated information is also displayed, if needed, including the one acquired by sensors deployed at farm level.

Additionally, new elements can be created within the GIS, such as polygons to identify regions of images or data views. Since all the information displayed by the GIS should be processed to extract knowledge useful for experts, a graphical editor has been added to help users to make calculations needed in the agronomic context. Basic operations are displayed as components in a graph that represents complex equations, often used to calculate relevant agronomic parameters. The outputs of these graphical equations are shown in the GIS interface and rendered in place. Furthermore, the operations described by these graphs could be properly serialized and exported in other formats or programming languages, if necessary.

The GIS tool groups the information by using layers, which are automatically rendered in the map and can be manually activated or deactivated. Type, size and source of data have influence on how the map is rendered, and even visualization can be temporally disabled. This flexibility allows data to be represented in a meaningful way and simplifies index calculation, for example NDVI or others.

In particular, Figure 5a shows an information layer calculated from a set of images collected by a drone on 15 August 2021, in a specific crop. Figure 5b displays an isoline map for cartographically showing temperature data collected on 8 August 2021.

### 2.7. Estimation of Irrigation Volume

At the beginning, the irrigation was scheduled according to the ETc determined by FAO methodology [26]. ETc was determined as the product of reference crop evapotranspiration (ET0), the crop coefficients proposed by the Agricultural Information System of Murcia (www.siam.es accessed on 20 November 2021) for this area. Later, the irrigation volumes were adjusted to maintain a certain soil water depletion in relation to the field capacity. This value ranged, depending on the crop phenological stage, between 5 and 20%, which the platform allowed to control by means of the graph of the soil water stock.

## 3. Results

This section describes the main tests and results obtained after deploying the Irriman Platform. First, the WEB application functionalities are evaluated, in depth. Then, Cloud interaction performance and computational results are analyzed. Finally, agronomic results are also highlighted.

### 3.1. Analysis of the Functionalities Provided by the WEB Application

**Figure 6:**
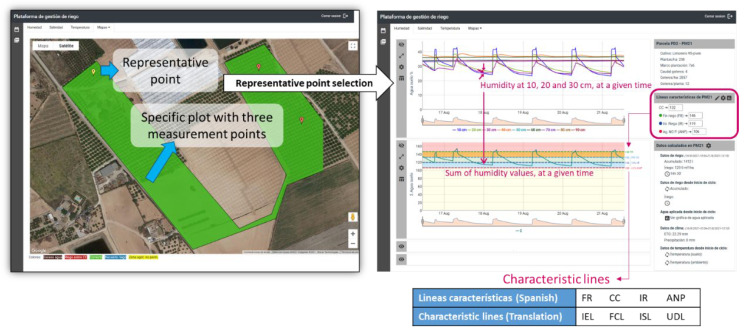
Representation of a specific plot. Three measurement points are defined. The plot’s representative point is marked with yellow color. For example, if the representative point is selected, data from the last week are downloaded and suitably represented by using charts. The second chart shows a time series defined as the sum of humidity values at depths of 10, 20 and 30 cm, since in this sample, the user had specifically selected such depths for the aggregation operation. Characteristic lines have been manually defined according to an agronomic expert’s knowledge.

As mentioned before, users access to the Irriman Platform through a WEB application and using the HTTPS protocol. Such application requests a unique login identifier and a password. If they are accepted, a validation string token is sent back allowing users to obtain the information of their owned plots. To activate the options of Humidity, Salinity and Temperature, and those in the lateral menu of the WEB application, a specific plot should be selected. Then, the map shows all the measurement points installed in the plot. When a measurement point is selected, timestamped sensor values are downloaded according to a range of dates (configured through the first option of the lateral menu), and it is possible to access to the graphic representation of humidity, salinity and temperature time series (see Figure 6).

A measurement point is a specific location in the plot where several sensors are installed. Such sensor channels could be part of different devices connected to the same or different data loggers. However, from the agronomic point of view, the measurement point is considered as an abstract concept, which provides information about the behavior of the soil, in a specific geolocated point in the map, through values of humidity, salinity, temperature and matric potential at different depths, independently of the electronic devices used for measuring them. As Figure 3 shows, the entity Measurement point has a complex property named “Additional features”, which stores several attributes for saving certain configuration data. There are four essential numerical configuration attributes: Irrigation End Line (IEL), Field Capacity Line (FCL), Irrigation Start Line (ISL) and Unacceptable Depletion Line (UDL). Such attributes are considered to be characteristic lines that agronomic experts define according to soil composition, type of crop and environmental conditions, and they allow farmers to know if the irrigation procedure is or is not suitable, by simply watching the chart that describes the evolution of the accumulated humidity value through time, calculated from humidity values obtained at different depths.

Note that, there is a representative measurement point defined in each plot, marked with yellow color on the map. Furthermore, the perimeter of the plot defines a polygon colored according to the legend defined in Figure 4. Such color is obtained from the values of the characteristic lines stored in the mentioned representative point, by calling the specific service (in the designed Endpoint), that takes the plot identifier as input and returns a code, which is converted to the corresponding color. The service accesses to the last humidity values (acquired during the last measured hour), at the different depths selected by the user (such selection is also saved in the Cloud, and it can be manually changed through the WEB application). It sums the values with the same timestamp, for each selected depth and, finally, it obtains a mean value from the resultant time series. If such value is under the UDL (the polygon is yellow), the crop is very poorly irrigated, and the plant could suffer dangerous water stress. If it is in the interval [ISL, UDL] (the polygon is blue), the crop is poorly irrigated, but water stress is still low. If it is in the interval [FCL, ISL] (the polygon is green), the crop is properly irrigated. If it is in the interval [IEL, FCL] (the polygon is red), it is necessary to stop irrigation if it is active, since the applied water has exceeded the field capacity. Finally, if it is over IEL (the polygon is dark red), the crop is highly overwatered and it could trigger the leaching process, which could contribute to polluting subjacent aquifers.

**Figure 7:**
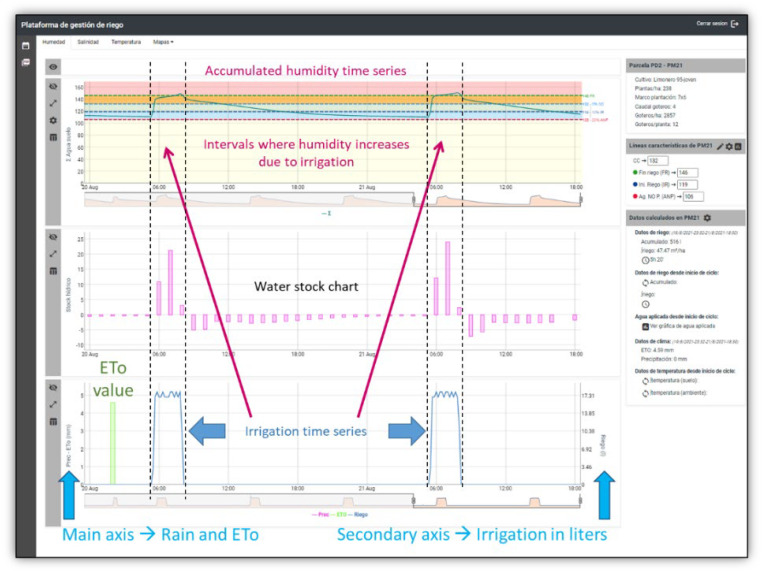
Other valuable charts shown when the Humidity tab is selected. In this case, the ET0 value is not available for 21 August, because the WEB application was accessed in such date. This happens since meteorological data (rain and ET0), specifically, one value per day, are provided by the external service IMIDA SIAM, which updates the information at the end of every day. Rain value is zero; otherwise, it would be represented by a bar such as the ET0 value.

Figure 7 shows the rest of charts which could be displayed together with the humidity and accumulated humidity time series: Water stock and Irrigation-Rain-ET0. Additionally, if matric potential time series is available, its chart is represented under the Irrigation-Rain-ET0 graphic. Furthermore, charts are synchronized with the aim of facilitating the understanding of the soil dynamics. Specifically, charts represent the acquired values taken during 20–21 August 2021.The irrigation procedure is started at 05:20 and it finishes at 08:00 every day. Water stock represents changes that happens in soil when water is provided. Increments are obtained while irrigation is active, and decrements occur when irrigation finishes. The accumulated humidity signal rapidly grows when irrigation starts, however, its fall is much slower. This depends on the soil dynamics, which is also reliant on environmental parameters (ambient temperature, for example), and crop type.

All this information and how it is represented is significantly illustrative for helping farmers to understand how irrigation is having influence in the soil and, consequently, in the plant. The expertise of agronomic engineers and technicians is necessary to manually define the characteristic lines; therefore, the Irriman Platform enables their configuration by allowing the values of such lines to be updated and stored in the Cloud. Once the lines are well defined, it is important to maintain the accumulated humidity signal in the range [FCL, ISL], as much as possible, by adapting the irrigation time and amount of added water. As mentioned before, values higher than IEL or lower than UDL could be reached. If such values stand, during a long period, in those levels, over IEL or under UDL, the leaching process could be triggered or water stress could affect plants. The first situation is environmentally harmful (for instance, in the context of protecting the aquifer connected with the “Mar Menor”), and the second one could lead to crop damage and reduce productivity. Therefore, the Irriman Platform offers a usable tool to track and control the irrigation procedure, since soil behavior (by considering different variables) is continuously monitored.

**Figure 8:**
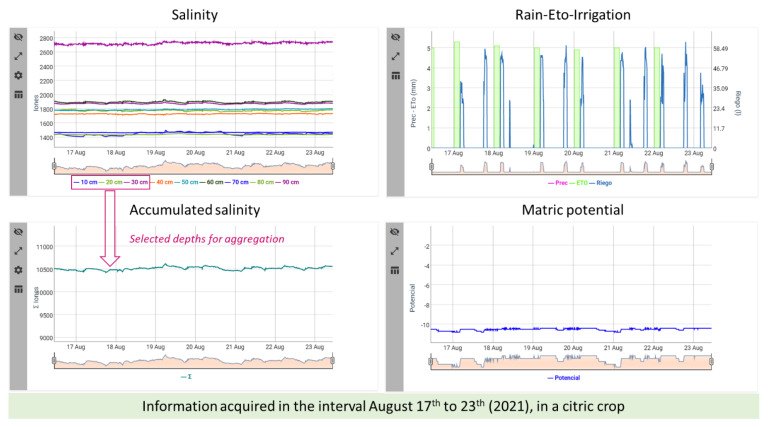
Charts shown when the Salinity tab is active. In this case, there exists a sensor capable of measuring matric potential, then, its time series is represented at the end of the char set. The accumulated salinity provides the time series resultant of value aggregation at 10, 20 and 30 cm.

Salinity and temperature could also be monitored. The Salinity tab shows the salinity chart representing time series at the same depths as humidity and temperature. In this case, the accumulated salinity is also plotted, by using the same aggregation algorithm than applied to humidity series; that is, the user makes a selection of depths through the WEB application (which can be registered in the Cloud), and adding the time series at the selected depths, a resultant time series is obtained and represented (see Figure 8).

**Figure 9:**
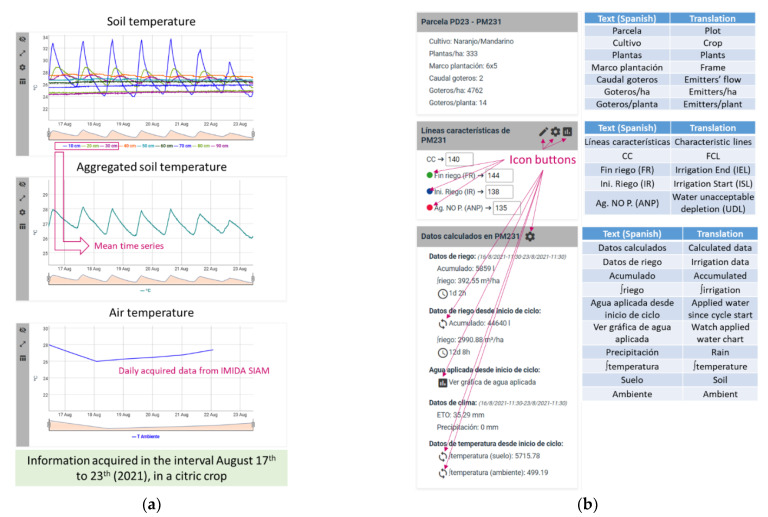
Information provided by the WEB application: (**a**) Charts shown when the Temperature tab is active. (**b**) Information regarding the plot. Text translation and icon buttons are highlighted.

Soil temperature information is also obtained from the same sensor device than humidity and salinity, consequently, there are time series at several depths. An aggregated set of temperature data (calculated as the mean of the values at different depths, previously selected by the user or stored in the Cloud through the WEB application), is also represented as a time series plotted together with the time series obtained from the external services provided by IMIDA SIAM, which offers daily information about the mean of air temperature (see Figure 9a).

The WEB application’s information cards show plot’s data and provide icon buttons that both, enable users to use the configuration tools and allow aggregation data calculation to be performed (see Figure 9b). For example, it is possible to easily configure the characteristic lines, to select the set of depths used for aggregation operations or to perform operations from the start of the crop cycle related to irrigation or temperature.

**Figure 10:**
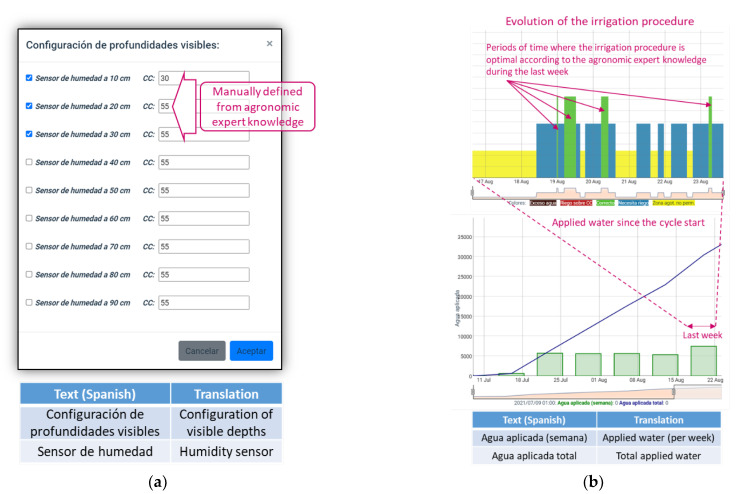
Other tools provided by the Irriman Platform. (**a**) Dialog for configuring the FC for each depth and for selecting which ones are active. (**b**) Charts for analyzing if the irrigation procedure is optimal.

The FCL value is calculated as the sum of all field capacities at different selected depths. For example, in Figure 9a, FCL is 140, since it is the sum of the values assigned to each field capacity (FC), used as inputs of the aggregation operation at 10, 20 and 30 cm. Such FCs are configured through the dialog shown in Figure 10a. When the agronomic expert uses the tool that allows the selection of active depths, he/she should define each FC value. Once the sum of FC values is updated, all the lines are erased, except the FCL. Then, the user should define the rest of the lines according to the value of FCL; thus, the order of the lines should be (from highest to lowest value) IEL, FCL, ISL, UDL.

It is also possible to track how irrigation is being applied, in a specific measurement point, through time. Figure 10b shows two specific charts for enabling this task. One chart allows users to analyze the evolution of the irrigation procedure according to the colors used for filling the polygon that defines a plot. Therefore, it is possible to know, at a glance, how long humidity is not represented by the green color, that is, out of the optimal interval [FCL, ISL]. The other chart shows aggregate information about how much water has been applied since the crop cycle start (represented as a line), and how much water has been applied each week (represented as bars).

Finally, as mentioned in Section 2.6, the GIS tool (see Figure 5) can be easily accessed from the WEB application and provides all the necessary functionalities for those users interested in including punctual measurements, manually taken through specific agronomic devices, together with information provided by drones and Hyperspectral Imaging satellites. Additionally, agronomic calculations could also be performed by using a graphical interface, which simplifies the edition of typical equations often used in the agronomic context.

### 3.2. Results Related to Cloud Computing Techniques

One of the main advantages of using Google Cloud Tools is that many analytics tools are freely provided for performance analysis. Some parameters have been measured in this work, for example, latency, number of GAE instances consumed, CPU (Central Processing Unit) utilization, number of sent and received bytes and amount of data stored in the Datastore. Thus, it is possible to analyze the global performance of the designed system. Furthermore, economic cost is also considered.

Instances classified as backend (B) and frontend (F) are the basic building blocks of App Engine. All the necessary resources are provided by them, and, at a given time, the application could be running on one or many instances with requests being spread across them. In addition, instance classes specify the amount of memory and CPU available to each instance, its amount of free quota and its cost per hour (once free quota exceeds). Nine instance classes are available: from F1 (default), to F4_1G for frontend instances and from B1 (default), to B8 for backend ones. The F1 class provides 120 MB of memory limit and a CPU limit of 600 MHz. This is valid for the first App Engine Standard Generation Runtime, which has been used for implementing the proposed system under Java 8.

**Figure 11:**
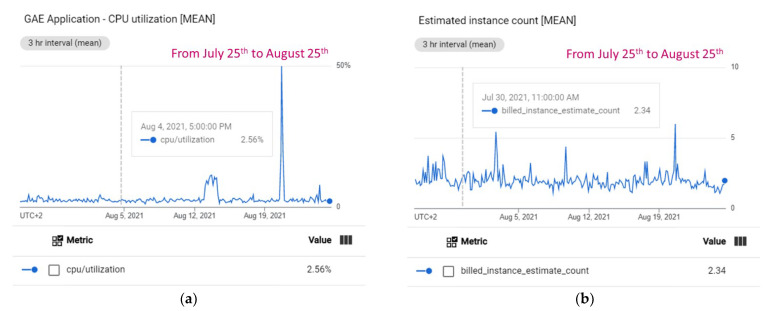
Performance measurements in a month. (**a**) CPU utilization during the analyzed period. (**b**) Estimated instance count during the evaluated period.

App Engine automatically creates and shuts down instances according to traffic fluctuations. There are several scaling types, controlling when instances should be created: automatic (by default, it is used in the proposed system), basic and manual. Only frontend instances support the automatic scaling type. Economic cost partially depends on scaling type, that is, the number of instances created for running the application. More instances usually involve better performance, but higher cost. The instance class also has influence in performance and cost. As mentioned above, the Irriman Platform uses the class established by default, then, the cost is USD 0.06 per hour per instance, since the selected region for deployment is “europe-west3”. As free quotas reset every 24 h, then, costs are only applied when quotas are exceeded in such period. For frontend instances the free quota is 28 h per day and for backend instances the free quota is reduced only to 9 h per day. Although the first version of the Irriman Platform was released in January 2021, and a major update was deployed in July 2021, a period of time of only one month (from 25 July to 25 August 2021), has been selected for performance evaluation. This period has been chosen, because several new plots were included in the platform after May 2021. Figure 11 shows information about CPU utilization and estimated instance count for such period.

Note that, the CPU utilization is near 3% almost all the time (see Figure 11a), and the estimated instance count is near 2.5% (see Figure 11b). This last value provides information about the mean number of billable instances. That means that instances have been billed because the free quota has been exceeded.

The GAE application provides services through an API REST by using Endpoints Framework. Such services are utilised by both the data loggers and the WEB application running simultaneously in different machines for diverse users. The main services are “savepaquetesonda_conriegobateria_d” and “get_serie_datos_v20_ds”. The first service is utilised by the firmware running in each data logger and it allows sensors’ information to be properly stored in the Datastore. Each data logger emits data each 10 min, approximately. The second service is utilised by the WEB application, when users download such stored information, a previous step before representing the time series in a specific measurement point. A JavaScript function performs the operation for downloading data from all the sensors in a specific measurement point, in a specific time range, by running a set of concurrent workers, each one of those call the service “get_serie_datos_v20_ds” for a specific sensor channel. Thus, if a measurement point includes, for example, a probe with 9 depths (for temperature, humidity and salinity), and an irrigation emitter is also installed, a total number of 28 sensor channels should be queried. Consequently, 28 calls to the service are performed in groups of 10 concurrent calls, since this is the maximum number or threads (workers), simultaneously created at the client side.

**Figure 12:**
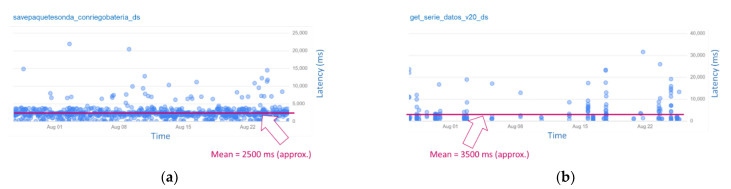
Latency charts for the main services. (**a**) Latency calculated for requests to the service used by data loggers to store sensor information. (**b**) Latency calculated for requests to the service used by users for downloading sensor information in a given sensor channel.

Latency statistics have been obtained for measuring how long these two services take for returning a response when they are utilised. Figure 12 shows that both spend a mean value lower 3500 approximately.

**Figure 13:**
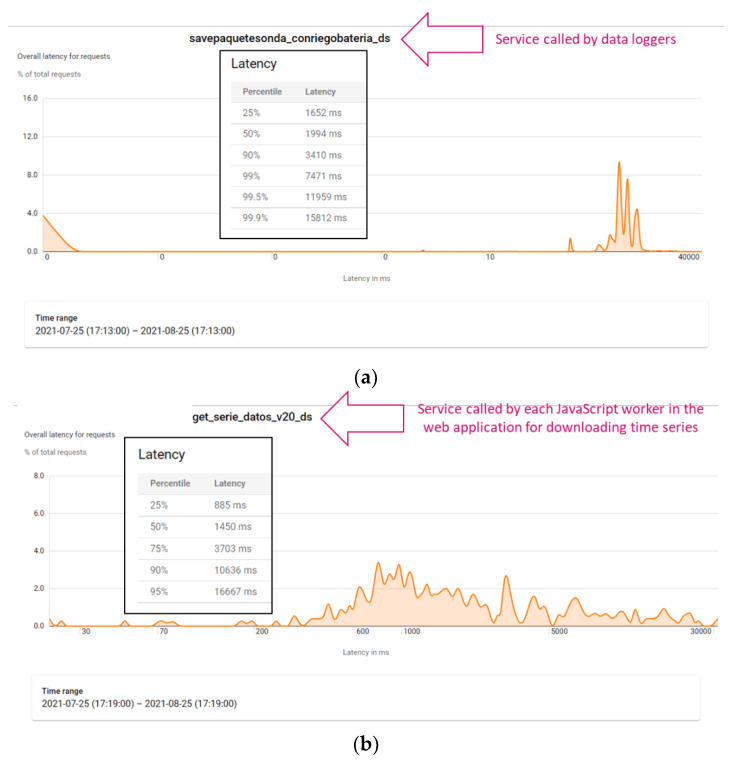
Distribution of the overall latency for request for the main used services. (**a**) Latency distribution for the service utilised by data loggers. (**b**) Latency distribution for the service used by each JavaScript worker in the WEB application.

Specifically, the service “savepaquetesonda_conriegobateria_d” returns a response fast enough (under 2500 ms on average), as Figure 12a shows, and the service “get_serie_datos_v20_ds” takes under 3500 ms (see Figure 12b). Note that, the service utilised by data loggers is used more frequently than the one used by users to download sensor data, because sensor data are sent all the time, but downloads are requested by users when they need them. Figure 13 shows the statistic distribution of the overall latency for requests for the mentioned services, in detail, during the evaluated period.

Figure 13a shows the percentiles, which provide more detailed information about the statistics distribution of the overall latency for “savepaquetesonda_conriegobateria_d”. All the requests take a time under 15812 ms and the 90% of them actually take less than 3410 ms. As strict real time processing is not necessary, latency is suitable for allowing data loggers to send data in a reliable manner by using the HTTPS protocol.

On the other hand, latency percentiles for “get_serie_datos_v20_ds” demonstrate that the 75% of requests take a time less than 3703 ms, and the 50% less than 1450 ms (see Figure 13b). This fact involves that if, in a measurement point, many sensors are installed (for instance, 28 sensors, as defined in the example mentioned above), and each request takes approximately 4 s, almost 2 min are needed to download the information. This also depends on the range of requested timestamps. In general, by default, data from the last week are downloaded for each measurement point (nevertheless, this range could be manually changed by one option available in the WEB application). Therefore, if values are acquired every 10 min, it is necessary to search and download 1008 entities of Stored value, per sensor channel. Consequently, downloading the weekly information for a measurement point with 28 sensors involves downloading 28224 entities of Stored value. To reduce download time, JavaScript workers are used. For instance, for the measurement point with 28 sensors taken as an example, two consecutive groups of 10 workers, which concurrently run, are created. Then, two remaining workers complete the download. Thus, global time is significantly reduced to 10 s or less for data in a range of a week, improving the usability of the WEB application.

**Figure 14:**
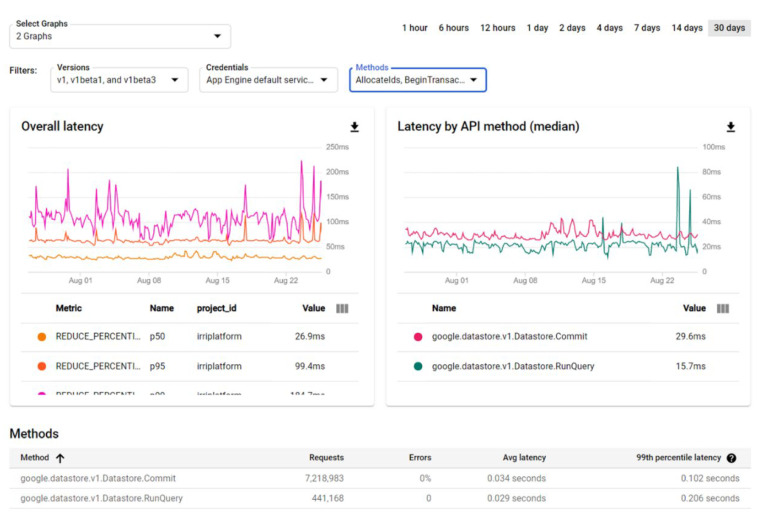
Results for latency when the requests to Commit and RunQuery methods are carried out.

Note that, when any service that needs to query, insert or update the Datastore is utilised, the total time spent for emitting the response includes different operations. Several of these operations are related to Datastore service usage through the Datastore API. Figure 14 shows the overall latency and the median of the latency values by API methods. In particular, a statistical measurement of the latency for *Commit* (related to database transaction operations) and *RunQuery* (related to database queries) methods is shown. The method *Commit*, with more than 7 million of requests carried out during the analyzed period, has an average latency of 0.024 s. More than 400,000 requests have also been carried out with the *RunQuery* method, with an average latency of 0.029 s.

**Figure 15:**
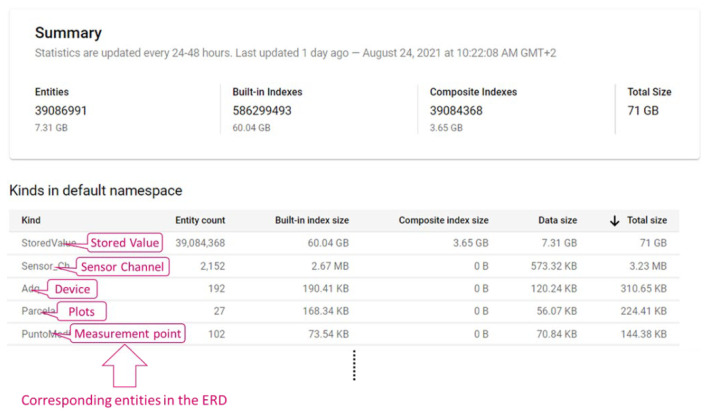
Summary of statistics about the use of the Datastore, while the application is running in the backend for all the users. Information about number of stored entities, data size and global size is automatically calculated.

**Figure 16:**
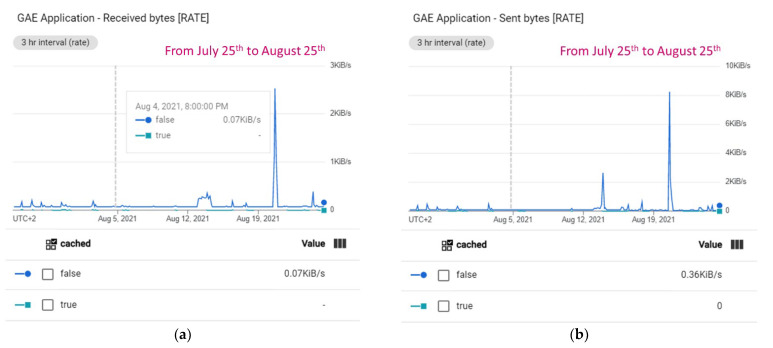
Other statistics related to byte rate which are received and sent when the application in the backend is running. (**a**) Received byte rate is under 0.25 KiB/s. (**b**) Sent byte rate is under 0.36 KiB/s.

**Figure 17:**
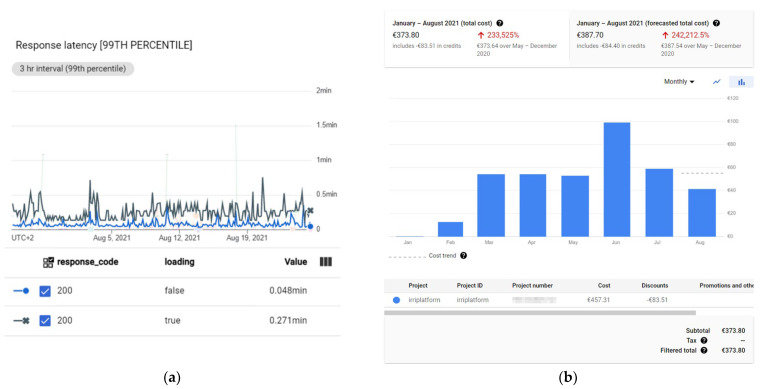
Statistics that summarize the response latency and the billing results. (**a**) Response latency for requests with a response code of 200. (**b**) Billing information calculated monthly.

Figure 15 shows the Datastore statistics calculated from the first deployment of the Irriman Platform to 24 August 2021. More than 39 million of entities have been stored up to this date (7.31 GB of agronomic information). In addition, Datastore has automatically built almost 600 million of indexes and near 400 million of composite indexes. Therefore, the total size consumed is 71 GB. Different plots have been progressively added to the proposed system. During the first half of 2021, 27 plots have been equipped with sensors, in particular, 2152 sensor channels in 102 measurement points with 192 installed devices. Clearly, more than 39 million of stored entities correspond to the stored value, with a size of 7.31 GB. The built-in index size for this kind is high (60.04 GB), because Google scales with data size. Figure 16 shows information about the rate of bytes that the GAE application has received and sent during the analyzed period. The rate of received bytes is mostly under 0.25 KiB/s (see Figure 16a), and the rate of sent bytes is under 0.36 KiB/s, in general (see Figure 16b). Figure 17 shows the response latency and information about billing during all the period since the first deployment of the proposed system.

In particular, Figure 17a shows the response latency for those HTTPS requests which returned a 200-response code (that is, the response indicating that the request has been properly processed). Billing information is monthly presented in Figure 17b. Note that the global cost of the Irriman Platform since it was deployed is EUR 373.80. This means that the average cost, in eight months, is EUR 46.72 per month. However, not all the months present the same cost, since this is dynamically calculated by Google according to the system usage. The peak in June is due to many real tests carried out for validating the Irriman Platform version update. The first months are cheaper because many plots were equipped with sensors and their information was included after February 2021.

If a value close to EUR 60 is used as the predicted monthly cost, it is possible to calculate the economic cost for each measurement point. There are currently 102 measurement points; consequently, the monthly cost is EUR 0.588 per point. If a measurement point includes approximately about 30 sensor channels, the cost per sensor would be EUR 0.019. Obviously, more measurement points with a high number of sensors increase the economic cost of monitoring a specific user’s plot. If a farmer wants to monitor his/her plot with three measurement points, for instance, with a total number of 90 sensor channels, the monthly cost would be EUR 1.71, and the consumption of Cloud resources would be EUR 20.52 per year. These numbers would be really increased, because they are evidently dependent on the number of requests carried out by users when they use the WEB application. However, Google Cloud allows developers to manage quotas and it is possible to analyze the use that each user makes of the proposed system. Thus, billing could be dynamically distributed between all the users according to their usage.

Any other systems that use a dedicated WEB server for implementing a similar application need to include the economic cost of system administration. Therefore, according to billing data, the use of the proposed system is clearly cheap, and even the global cost for farmers would also be reduced, since plot monitoring helps them to save water and other inputs and the use of Irriman Platform contributes to optimize the irrigation procedure according to environmental needs, such as those related to ecosystem protection, for example the “Mar Menor” in the Region of Murcia, Spain.

### 3.3. Agronomic Results

**Figure 18:**
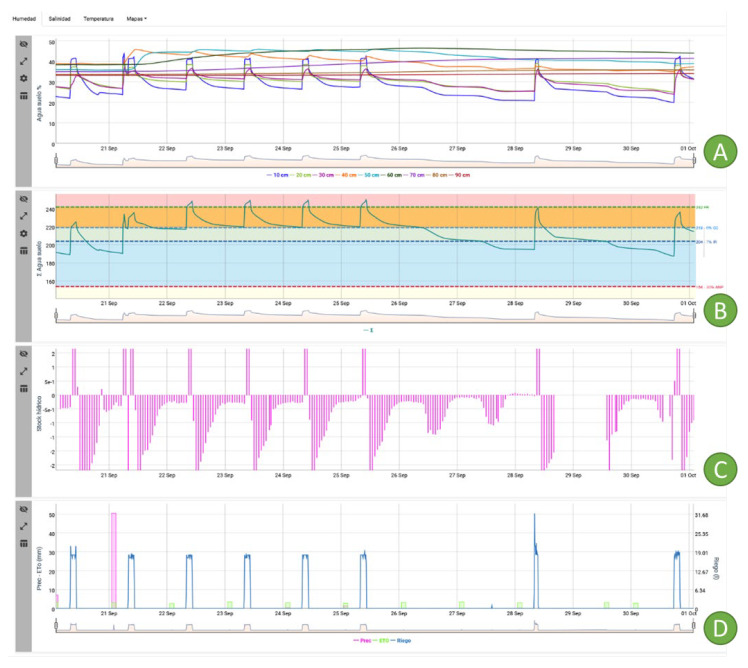
Evolution of the soil volumetric water content at different depths (10 to 90 cm) in the adult lemon tree crop (**A**). Sum of the volumetric water content in the soil at the depths of maximum root density (**B**). Variation of the volumetric water content in the soil for two consecutive time instants (**C**). Values of water applied through irrigation, evapotranspiration of the reference crop and rainfall during the period shown from 20 September to 1 October 2021 (**D**).

Figure 18A shows the evolution of the soil volumetric water content at different depths (from 10 to 60 cm) during the period from 20 September to 1 October, in an adult lemon tree located in Campo de Cartagena (Murcia, GPS (37.673, −0.996)). This is one of the sensorised demonstration plots of the platform. The shallowest sensors—in the 0 to 40 cm depth profile—detected the irrigation applied (Figure 18D), as can be seen on 20 September for an irrigation time of 2 h 30 min (applied in the morning). However, the rainfall of 52 mm on 21 September increased the soil moisture in the entire soil profile, immediately up to a depth of 60 cm, and more gradually (visible after the end of the rain) up to 90 cm. Subsequently, the daily irrigation time was maintained at the same level as before the rain, significantly increasing the water content in the soil profile (21–25 September), until irrigation was stopped for 3 days until 28 September. This led to a decrease in soil moisture in the soil profile down to a depth of 40 cm, to pre-rainfall levels (Figure 18A).

From this moment on, the interval between irrigations was extended to 2–3 days, even with a reduction in the irrigation time to 1 h 30 min (Figure 18D). The rainfall on 21 September facilitated the definition of the so-called soil water characteristic lines, which are specific to each farm and crop, in this case, and given the effective depth of its root system, from the sum of the sensors located in the profile from 0 to 60 cm (Figure 18A,B).

Thus, the field capacity was detected (blue dotted line) 24 h after the rain, after leaching of the drainage water, and served as a reference to establish the line of maximum permissible depletion (dark blue dotted line), which refers to the percentage reduction in water content with respect to field capacity, whose value for the phenological stage in which this crop was found was set at 7–10%. The lower limit of this reduction in moisture content would be established by the red dotted line, which defines the maximum permissible depletion (Figure 18B). The green dotted line would allow the maximum irrigation time to be set to avoid leaching of water and nutrients provided by irrigation below a certain depth, in this case 60 cm deep. Finally, Figure 18C corresponds to the hourly depletion of water in the soil, and its values can be compared with the root activity with respect to the absorption of water in the soil; thus, on 25 and 28 September, it can be seen that the area delimited by the pink bars corresponding to day 27 is lower than that of the previous day, a sign that reflects a lower absorption of water by the roots, and indicates the time of the start of irrigation.

## 4. Discussion

Irrigation scheduling based on the knowledge of the soil water status has been validated by numerous investigations in different crops [27,28]. In our case, the integration of data from these sensors in a platform has provided the farmer with a more sustainable irrigation control, reducing the frequency and time of irrigation after the rain of 21 September (Figure 18D). In this way, knowledge of the soil water status of the farm in real time has made it possible to wet the soil profile only explored by the roots of the crop grown on the farm, limiting the leaching of water and nutrients and the possible contamination of aquifers [29,30].

Irrigation scheduling based on the maintenance of water in the soil according to the definition of the characteristic lines of water in the soil, has also allowed us in semi-arid conditions to optimize irrigation water by controlling the irrigation time, and even to implement a certain water deficit in the soil, without exceeding the maximum permissible depletion of water in the soil. In the example case, water in the soil has been reduced by up to 7% compared with the field capacity (Figure 18B), by reducing the irrigation time and decreasing the irrigation frequency. In this way, the water stress to which the crop is to be subjected is controlled, according to the phenological phase in which it is found, i.e., it is possible to schedule the regulated deficit irrigation in real time and to establish more accurately a threshold value that would correspond to a water stress suitable for the crop, to avoid losses in the production and quality of the harvests [22,31,32].

From the technical point of view, the use of Cloud tools for software architecture implementation and deployment offers advantages if the system is compared with those that use classical deployment options based on using typical WEB hosting systems. On the other hand, if a workstation is acquired and properly configured for providing WEB services, it is necessary to hire technical personnel to carry out all the tasks related to system management and, consequently, it is necessary to consider the economic cost of the staff needed for system maintenance.

Farmers and other stakeholders need to access the system easily, and the number of potential customers could exponentially grow if many plots are included in the platform. Therefore, a solution based on the acquisition of a workstation or on the usage of typical hosting systems for storing all the software components and data are not enough, since scalability is required for the Irriman Platform to properly work. Many devices simultaneously interact with the backend, and many users could be using the WEB application to monitor their crops; then, it is necessary that the infrastructure which hosts the backend can easily carry out the requested scaling tasks.

The Irriman Platform uses the standard App Engine environment, which facilitates the building and deployment of highly scalable applications that run reliably even under heavy load and with large amounts of data, on a fully managed serverless platform. Consequently, there is no need to carry out infrastructure management tasks; that is, there is no server management and configuration.

Google offers a flexible payment method based on usage. If free quotas are not exceeded, there is no need to pay anything, but if such quotas are exceeded, it is necessary to pay only for the exceeded resource. This is an advantage in comparison with providers that require a flat rate if only a certain number of quotas are known to be exceeded.

For each user (for example a farmer), it would be possible to establish a specific economic plan based on subscription accounts. Note that, according to Section 3.2, for a plot with three measurement points, with a total number of 90 sensor channels, the monthly cost of storing the sensed data would be EUR 1.71. This cost would be lightly incremented with the use of the WEB application. Therefore, the global economic cost is very competitive.

The services offered by Google have been demonstrated to be highly available during a long period of time. That means that the Irriman Platform is very reliable, and data loss is practically null. Moreover, Google claims that it is a carbon neutral company with the aim of reaching the goal of running on carbon-free energy, all the time, at all their data centers by 2030. In fact, it currently includes an integrated tool for measuring the carbon footprint of any Cloud-based application, which allows developers to view the gross, location-based emissions that derive from the Google Cloud usage. This is clearly an advantage in comparison with other solutions.

Although, there exist companies which have developed commercial software tools for helping to monitor crops, many of them only use hyperspectral visual information from satellites or other partial information, but they do not usually combine a wide range of measurements, coming from many different sources, such as the Irriman Platform does. Furthermore, other monitoring tools existing in the market are totally closed solutions, whereas the Irriman Platform is based on a distributed architecture which could be easily extended if required. Consequently, the proposed system provides advantages with respect to the existing ones, both in the research and commercial contexts.

Finally, as agronomic results show, the Irriman Platform could be considered as an essential tool for agronomic experts in the Cloud (with all the advantages that this fact assumes), such as it provides usable options through a WEB application for analyzing and configuring the irrigation strategies in sensorised crops, focused on reducing water usage and protecting environmental ecosystems from pollution due to leaching process. However, equipping crops with soil sensors requires a starting investment and continuous maintenance. Many farmers are not equipping their crops with this kind of technologies, since the economic cost of certain sensors could be high. This situation is being taken into account in a new enhanced version of the system. In fact, several plots currently included in the Irriman Platform are considered as “demonstrative plots”. It is expected that they allow agronomic experts to analyze the relationship existing between data gathered by soil sensors and calculated indexes obtained from the acquired multispectral and hyperspectral images [33]. If such relationship is found, it would be possible to design irrigation procedures only using images by inferring values, typically collected by soil sensors, from such images. Thus, if there exists an optimal irrigation procedure applied to a demonstrative plot, it could be transferred to plots not equipped with such sensors, but with a similar soil behavior, by simply considering indexes obtained from images. In addition, the availability of real-time data for different crops and soils allows the implementation of predictive models to know the most suitable moment to apply water to the crop [34].

On the other hand, the automation of the whole process for applying an irrigation strategy is also planned and it will be implemented in the new version of the Irriman Platform. In this case, actuation will also be automatically performed, according to the inputs provided by the agronomic experts, who will be advised by a DSS, which will allow soil dynamic behavior to be predicted under different meteorological and irrigation conditions, by using a soil model obtained through Machine Learning techniques.

## 5. Conclusions

Nowadays, we are faced with an ever-increasing need to feed a population that is constantly growing, and which is also more demanding in terms of quality and environmental sustainability. For this reason, it is essential to have adequate soil water monitoring that informs us of the soil and crop water status in real time, so that we can adapt the irrigation time to the capacity of the soil to retain the water applied, mitigating or avoiding the leaching of water and nutrients. Thus, the resulting agriculture would be more sustainable, not only being environmentally friendly, but also more economically viable, as it would be more competitive, by reducing water, nutrients and energy, as the irrigation systems are pressurised.

The Irriman Platform clearly contributes to this objective, since it allows agronomic experts, farmers and other stakeholders (even potential customers of agricultural products) to track if production is sustainable from an environmental point of view, and if it is focused on reducing or even avoiding aquifer pollution. The proposed system offers a set of Cloud-based services through a WEB application, which different stakeholders can use according to their roles. Such services are implemented as a set of distributed software components in an application running in the App Engine Standard environment. The data model is implemented as a NoSQL database by using Google Firestore in Datastore mode, with automatic scaling, high performance and ease of application development with a strongly consistent storage layer and real-time updates, ensuring a safe and private storage system, since information is properly encrypted. Data loggers implement a low-level software capable of easily interacting with the Cloud services by sharing JSON messages, by considering important issues as reliability in measurements and low energy consumption. Furthermore, a wide set of data sources is allowed, including external information provided by national and local institutions such as the IMIDA SIAM net of stations.

The Irriman Platform is currently deployed in a wide number of crops in the Region of Murcia, with a low economic cost. It has been uninterruptedly working for more than a year, demonstrating that it is possible to reliably acquire and store data gathered at soil level, external environmental information from IMIDA SIAM and hyperspectral and multispectral information from satellites and drones, respectively. All the information is properly processed and presented to farmers, agronomic experts and other stakeholders by using a WEB application, with the aim of enabling them to design environmentally sustainable irrigation techniques that provide good results in terms of saving water and avoiding the leaching process.

## Figures and Tables

**Table 1:** List of agronomic sensors currently deployed in the plots monitored by the Irriman Platform.

Agronomic Sensor	Description
Drill and Drop	Soil water content, temperature and salinity probe
MPS6	Device for measuring soil matric potential and soil temperature
Flow meter	Applied irrigation measurement

## Data Availability

Not applicable.
